# Thulium YAG laser versus bipolar enucleation for management of benign prostate obstruction secondary to large prostates (>80 gm): A multicenter prospective randomized study

**DOI:** 10.1080/20905998.2025.2509042

**Published:** 2025-05-22

**Authors:** H. Shaker, A. Yehia, M. El Adawy, Mohamed Abd El Ghani, A. Kassem, M. Abd El Hamid

**Affiliations:** a Urology Department, Fayoum University, Egypt; b Urology Department, Cairo University, Egypt

**Keywords:** BPH, enucleation, bipolar, THULEP, laser

## Abstract

**Objectives:**

Endoscopic enucleation of the prostate (EEP) was introduced to treat patients with large prostate. The study compared the perioperative and post-operative outcomes of bipolar current and Thulium-YAG in endoscopic enucleation of large prostates above 80 gm.

**Patients and methods:**

This is a prospective study conducted on 120 male patients. The patients were randomized into two equal groups; Group A Thulium laser enucleation of the prostate (THuLEP) and Group B bipolar enucleation of the prostate (BEEP). All patients were preoperatively evaluated as regards operative time, hemoglobin drop, intraoperative and early postoperative complications, hospital stay, and time of catheter removal. Patients were then followed up at 1, 3, 6, and 12 months to detect urinary and sexual functional outcome and any postoperative complications.

**Results:**

The preoperative characteristics of both groups were comparable. The mean prostate volume was 122.33 ± 24.34 (84–180 gms) and 120.88 ± 25.66 (85-180gms) (*p* value: 0.751) in THuLEP and bipolar groups, respectively. Significant comparable improvement in IPSS score, Q max, and PVR postoperatively was found in both groups at all follow up points. Follow up after 1 month revealed urgency urinary incontinence in 34 (56.7%) cases in THuLEP and 14 (23.3%) cases in bipolar enucleation group (*p* value 0.001), and stress urinary incontinence was detected in 44 (73.3%) cases in THuLEP and 26 (43.3%) cases in bipolar enucleation group, respectively (*p* value 0.001). Both types of incontinence improved after 3 months in both groups, three cases in the THuLEP group had persistent stress incontinence after 1 year.

**Conclusion:**

ThuLEP and bipolar enucleation are comparable treatment modalities for patients with large prostate (>80 ml).

## Introduction

For a long time, ‘Trans urethral resection of prostate (TURP)’ was the standard of care modality for prostate lesser than 80 gm and open prostatectomy for prostate larger than 80 gm [1].

The term endoscopic enucleation of the prostate (EEP) was introduced to treat patients with large prostate. In 1986, Y. Hiraoka introduced endoscopic enucleation for the first time, where a monopolar detachment probe was used [2].

In 2010, Herrmann introduced the term anatomical enucleation of the prostate using Thulium laser (ThuLEP) [3,4]. ThuLEP exhibited equivalent safety concerning local complications and demonstrated similar effectiveness in comparison to the gold standard TURP [5].

Besides the shallow penetration depth (0.2 mm) Thulium YAG laser provides a continuous-wave pattern and, consequently, an easier-to-learn prostate enucleation technique with a shorter learning curve in comparison to holmium laser enucleation of the prostate [6].

BEEP is a good alternative to TURP, surpassing both bipolar and monopolar TURP in terms of morbidity and the length of hospital stay [5].

Studies comparing BEEP and ThuLEP as regard peri operative data are still scarce.

This study aims to compare bipolar current and Thulium-YAG laser in endoscopic enucleation of large prostates above 80 gm. The primary end point of the study is as follows: mean enucleation time for THuLEP in comparison to BEEP, while the secondary end point of the study was improvements in Qmax, IPSS, PVR, IIEF-5, SUI assessed by ICIQ-SF & complication rates following both techniques

## Patients and methods

A prospective controlled study conducted at urology departments, Cairo University & Fayoum University hospitals from the period of November 2022 to November 2023. A written informed consent was obtained from all the patients. The study was conducted with the principles of the Declaration of Helsinki and approved by the ethical committee of Fayoum University, &; (registered under IRB No.R349). The study was carried out in line with the Code of Ethics of the World Medical Association for research involving humans.

### Patient selection, sample size calculation and preoperative assessment

The study was proposed to include 110 patients (divided into 2 groups 55 in each group). The sample size was calculated according to the mean enucleation time for ThuLEP [7] 70.5 (58–87.3) min and BPEP 56 (23–75) minutes [8] Using Student's t-test with a 95% power and 5% α‐error level, the calculated sample size was 51 patients in each group. For the expected dropout during follow-up, we increased the sample size by 15% (60 patients in each group). Calculations were done using the G*Power 3.1.9.7 software power and sample size programme. *and chosen test was t-test (means: difference between two independent means (two groups).* A statistician randomized the eligible patients using block randomization and sealed envelopes. The participants were blinded to the assigned type of intervention at the time of surgery.

Inclusion criteria were male patients above 50 years of age, presented with lower urinary tract symptoms (LUTS) with prostate size of more than 80 gm by transrectal ultrasound (TRUS), refractory to medical therapy (a maximal flow rate (Qmax) less than 15 and international prostate symptom score (IPSS) more than 18), and/or developed complications of bladder outlet obstruction (BOO) as refractory hematuria, refractory urine retention, recurrent attacks of urinary tract infection (UTI), or upper tract affection.

Exclusion criteria included patients on anticoagulant or antiplatelet medications, those with prostate and bladder cancer, previous urethral/prostatic surgery, urethral strictures, or those diagnosed with detrusor underactivity by urodynamic study.

Preoperative evaluation included full history taking, digital rectal examination (DRE), international prostate symptom score (IPSS), IIEF-5 (The International Index of Erectile Function) score, urine analysis and culture, hematocrit concentration, serum PSA, trans-rectal ultrasonography (TRUS), uroflow, and abdomino-pelvic ultrasound and post void residual urine (PVR).

### Surgical technique and equipment

ThuLEP was performed by two surgeons who had performed more than 200 ThuLEP cases. Thulium YAG laser enucleation procedures were carried out using Revolix DUO ® Thulium laser unit (Lisa laser, Katlenburg-Lindau, Germany), power settings were 80 W for enucleation and 60 W for coagulation. The laser energy was delivered through an optical-core, bare-ended, reusable 550 μm RigiFib (Lisa laser, Katlenburg-Lindau, Germany). Bipolar enucleation procedures were done by the same surgeons using the Autocon II 400 SCB bipolar resectoscope (Karl Storz, Germany) and its enucleation loop. The resectoscope was a 26 Ch calibre with continuous irrigation (Karl Storz, Tuttlingen, Germany), and the morcellation was accomplished by Storz morcellator (Karl Storz GmbH & Co., Tuttlingen, Germany) which inserted by means of a nephroscope sheath in all procedures. Patients were blinded to the type of intervention as well as the statistician and data collectors.

The enucleation procedure, be it the two-lobe or three-lobe technique, was consistently utilized in both groups, ensuring early release of the prostate apex to prevent traction on the striated sphincter. Deep incisions were made into the surgical capsule at the 5 and 7 o’clock positions of the bladder neck, extending up to the level just proximal to the verumontanum. The enucleation of the median lobe commenced from the convergence of these two incisions at the verumontanum, with the sheath of the resectoscope bluntly pushing the tissue along the surgical capsule towards the 6 o’clock direction of the bladder neck. For the lateral lobes, the resectoscope was positioned at 12 o’clock, making an incision in the anterior commissure to create a furrow for enucleating the upper portion of the lateral lobes. The lateral plane was subsequently developed from the apex of the lobes to the 3 and 9 o’clock positions for the right and left lateral lobes, respectively, involving blunt lifting towards the bladder neck. Physiologic saline was used throughout the procedure. Following adequate haemostasis, the lobes pushed into the bladder were morcellated into small chips. All procedures were carried out under spinal anesthesia using normal saline. After adequate hemostasis, a 22 F three-way urethral catheter was fixed with continuous bladder irrigation by normal saline. Urethral catheter was planned to be removed on the first postoperative day unless indicated for any other reason such as hematuria or intraoperative complications. The patients were discharged after being able to void adequately without significant hematuria.

Operative time (enucleation time and morcellation time), weight of resected tissues, hemoglobin and hematocrit drop and need for blood transfusion, intraoperative and early postoperative complications, hospital stay, and time of catheter removal were monitored carefully.

All patients were followed up 1 week postoperatively for any early postoperative complications. All patients were asked to visit outpatient clinic at 1, 3, 6, and 12 months to detect urinary and sexual functional outcome by pelvic ultrasound for post-voiding residual urine (PVR), IPSS score, IIEF-5 score, International Consultation on Incontinence Questionnaire (ICIQ-SF), and Qmax. We also monitored any postoperative complications (according to Clavien classification) or any other urinary symptoms.

The authors confirm the availability of, and access to, all original data reported in this study

### Statistical analysis

Data were coded and entered using the statistical package for the Social Sciences (SPSS) version 28 (IBM Corp., Armonk, NY, USA). Data was summarized using mean and standard deviation for quantitative variables and frequencies (number of cases) and relative frequencies (percentages) for categorical variables. Comparisons between the groups were done using unpaired t test [9]. For comparing categorical data, Chi square (χ2) test was performed. Exact test was used instead when the expected frequency is less than 5 [10]. P-values less than 0.05 were considered as statistically significant.

## Results

**Figure 1.**
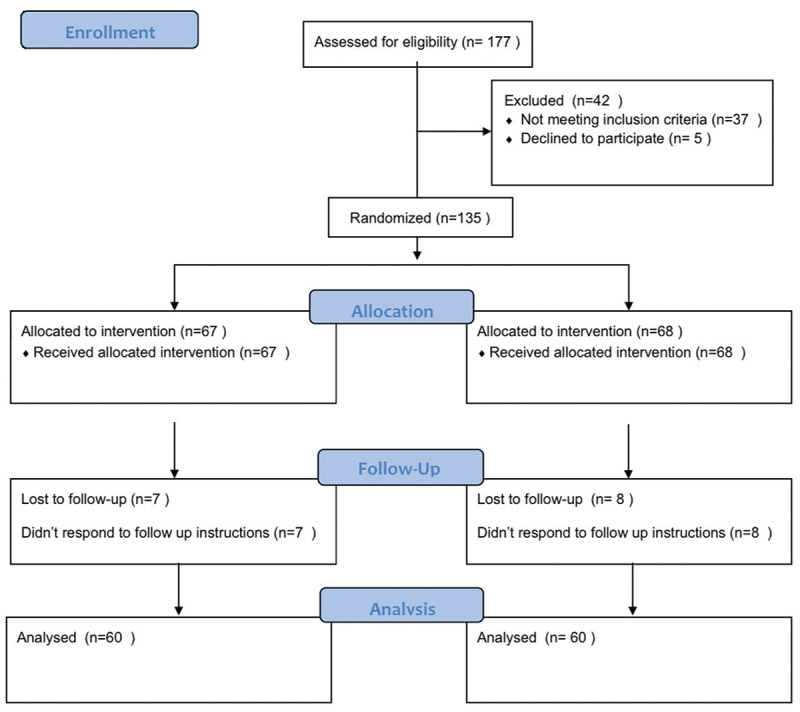
CONSORT flow diagram.

A total of 177 patients were enrolled in the study, 37 patients did not meet the inclusion criteria and 5 patients declined to participate in the study. Seven and eight patients, respectively, did not complete follow up in THuLEP and bipolar enucleation groups. So, 120 patients completed follow up. Sixty patients in each group. (Figure 1)

The preoperative data of both groups were comparable. The mean prostate volume in the ThuLEP group was 122.33 ± 24.34, median 120.50, range (84-180 gms) and in the bipolar group was 120.88 ± 25.66, median 117 gms, range (85–180 gms) (*p* value: 0.751) No significant differences between the two groups regarding mean age, preoperative IPSS, Q max, PVR, IIEF-5, PSA or Hb (Table 1).

**Table 1.** Indications, pre-operative and operative criteria of both groups.

		BEEP	ThuLEP	P value
Age		67.37 ± 5.95	69.50 ± 5.18	0.296
IPSS		25.68 ± 2.05	25.85 ± 2.17	0.691
PVR		129.85 ± 25.40	124.17 ± 24.48	0.275
Qmax (ml/sec)		6.75 ± 2.01	7.31 ± 2.26	0.208
Prostate size	Mean ± SD	120.88 ± 25.66	122.33 ± 24.34	0.751
	Median(range)	117.00 (85.00–180.00)	120.5 (84.00–180.00)	
PSA (Pre)		4.43 ± 1.26	4.72 ± 3.46	0.531
IIEF-5 (Pre)		15.20 ± 1.82	15.08 ± 1.89	0.731
Hb (pre)		13.07 ± 1.34	13.38 ± 1.34	0.211
Indications	**bladder stones**	5 (8.3%)	4 (6.7%)	1
	**failed medical TT**	36 (60%)	37 (61.7%)	
	**Hematuria**	6 (10%)	5 (8.3%)	
	**refractory retention**	13(21.7%)	14 (23.3%)	
Operative time		109.4 ± 11.79	113.4 ± 16.34	**0.12**
Enucleation time		79.68 ± 7.24	81.25 ± 39.76	**0.31**
Resected tissues (g)		102 ± 3.24	98 ± 7.34	**0.23**
Enucleation efficiency in gm/min		1.78 ± 0.37	1.88 ± 0.28	**0.089**
Morcellation efficiency in gm/min		5.18 ± 1.33	4.84 ± 0.96	**0.115**
Irrigation fluids (liters)		37 ± 2.7	40 ± 2.4	**0.09**
Hb drop (g\dl)		1.2 ± 0.1	0.9 ± 0.2	**0.165**
HCT pre		44.20 ± 2.92	44.60 ± 2.27	0.404
HCT post		41.05 ± 2.32	41.18 ± 2.16	0.745
HCT drop		3.15 ± 2.15	3.42 ± 1.29	0.413

IPSS: international prostate symptom score, PVR: Post voiding residual urine, Q max: Maximum flow rate, PSA: Prostatic specific antigen, IIEF-5: The International Index of Erectile Function.

The primary end point of the study was mean enucleation time. The mean operative time was (113.4 ± 16.34 and 109.4 ± 11.79, *p* value = 0.12), and the mean enucleation time was (81.25 ± 39.76 and 79.68 ± 7.24, *p* value = 0.31) in ThuLEP and bipolar groups, respectively, with no statistically significant difference between both groups in that. Mean enucleation efficiency was 1.88 gm/min for the THuLEP group, and 1.78 gm/min for the BEEP group, while the mean morcellation efficiency was 4.84 gm/min for the THuLEP group, and 5.18 gm/min for the BEEP group (Table 1), there was no significant difference between both groups regarding indications of intervention and total operative time.

There were no significant differences between both groups regarding hospital stay, time of catheter removal, resected volume and intraoperative blood loss expressed by hemoglobin drop (Table 1) Two patients in each group needed blood transfusion due to significant intraoperative bleeding (Table 2).

**Table 2.** Intraoperative and post-operative complications.

Clavien–Dindo classification	Bipolar	ThuLEP	P value	Study population
**Grade 1 (deviation from the normal course without the need for pharmacological treatment or surgical interventions)**				
Morcellation injury	0 (0.0%)	1 (1.7%)	o.98	1 (0.84%)
Capsular perforation	3 (5.0%)	4 (6.7%)	0.85	7 (5.8%)
Mild subtrigonal dissection	1 (1.7%)	1 (1.7%)	1	2 (1.7%)
**Grade 2 (pharmacological management)**				
Febrile UTI	2 (3.3%)	2 (3.3%)	0.82	5 (4.2%)
Orchitis	1 (1.7%)	1 (1.7%)	1	2 (1.7%)
Postoperative hematuria	2 (3.3%)	1 (1.7%)	1	3 (2.5%)
**Grade 3B (procedure under regional or general anesthesia)**				
Bladder neck contracture	0 (0.0%)	1 (1.7%)	0.87	1 (0.84%)
Stricture urethra	1 (1.7%)	0 (0.0%)	o.87	1 (0.84%)

UTI : Urinary tract infection.

**Figure 2.**
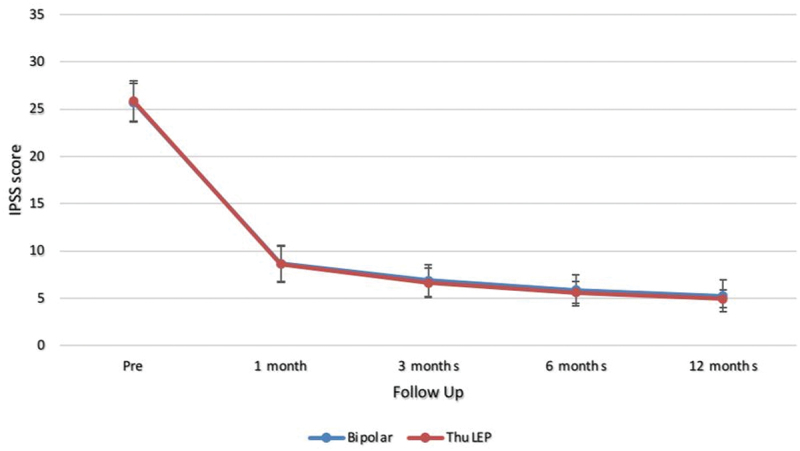
IPSS score line graph.

**Figure 3.**
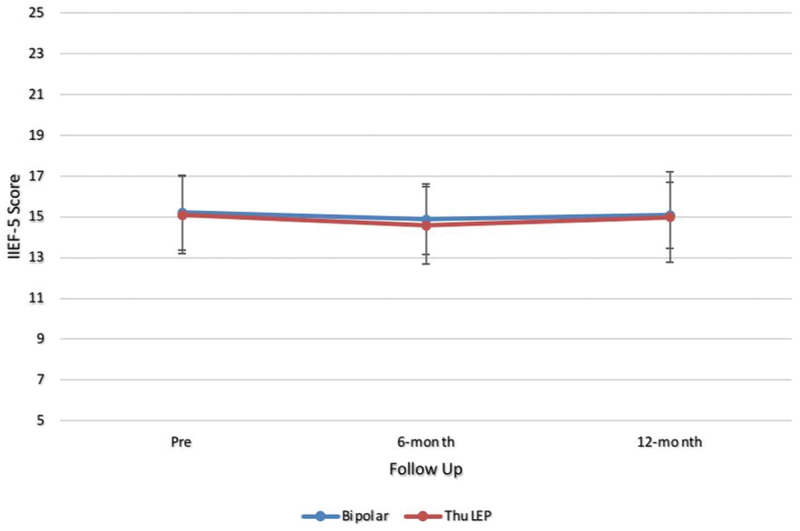
IIEF-5 score line graph.

Postoperative significant comparable improvement in IPSS score, urinary Q max, and PVR was detected in both groups at all follow up points. On the other side, there were no differences in Q max, IPSS and PVR between both groups at different follow up points either in postoperative values or in the percentage of improvement from baseline measures. At 1 year follow up, Q max was (34.93 ± 4.00 and 33.82 ± 4.97 *p* value: 0.423), IPSS was (4.95 ± 0.93 and 5.23 ± 1.69 *p* value 0.621), and PVR was (12.50 ± 3.95 and 13.43 ± 4.62 *p* value 0.236) in ThuLEP and bipolar group, respectively. Mean IIEF-5 was 14.98 ± 2.21 in ThuLEP and 15.07 ± 1.60 in bipolar group at 1 year with no significant difference (*p* value 0.313) (Table 3). Figures 2 and 3 show linear curves for changes in IPSS & IIEF over the period of follow up (Figures 2 and 3).

**Table 3.** Post-operative efficacy.

Parameter		Pre	1 month	3 months	6 months	12 months	P value
IPSS	Bipolar	25.68 ± 2.05	8.67 ± 1.96	6.88 ± 1.70	5.85 ± 1.64	5.23 ± 1.69	<0.001
	ThuLEP	25.85 ± 2.17	8.62 ± 1.87	6.63 ± 1.53	5.62 ± 1.11	4.95 ± 0.93	
	P value	0.691	0.887	0.398	0.231	0.621	
Q max	Bipolar	6.75 ± 2.01	26.43 ± 4.21	30.33 ± 4.14	32.30 ± 5.10	33.82 ± 4.97	<0.001
	ThuLEP	7.31 ± 2.26	27.23 ± 3.89	31.13 ± 4.83	33.65 ± 3.91	34.93 ± 4.00	
	P value	0.208	0.282	0.332	0.456	0.423	
PVRU	Bipolar	129.85 ± 25.40	34.67 ± 10.60	22.20 ± 6.00	17.47 ± 5.94	13.43 ± 4.62	<0.001
	ThuLEP	124.17 ± 24.48	32.87 ± 8.72	22.25 ± 5.24	16.20 ± 4.05	12.50 ± 3.95	
	P value	0.275	0.312	0.961	0.175	0.236	
**IIEF-5**	Bipolar	15.20 ± 1.82			14.87 ± 1.73	15.07 ± 1.60	0.356
	ThuLEP	15.08 ± 1.89			14.57 ± 1.89	14.98 ± 2.21	0.427
	P value	0.731			0.366	0.313	

IPSS: international prostate symptom score, PVR: Post voiding residual urine, Q max: Maximum flow rate, PSA: Prostatic specific antigen, IIEF-5: The International Index of Erectile Function.

For catheter-dependent patients, THuLEP offered higher Qmax values at 3 months and 1 year postoperatively, with statistically significant differences compared to BEEP. On the other hand, BEEP showed higher PVR values at 1, and 3 months postoperatively, with significant differences, while there is no significant difference at 1 year. Meanwhile, for catheter-independent patients, THuLEP showed higher Qmax values at 3 months and 1 year, with statistically significant differences compared to BEEP. While, BEEP showed lower PVR values at Month 1, with a statistically significant difference, yet other time points showed no significant differences between both modalities (Table 6).

**Table 6.** Improvements in Qmax & PVR for catheterized and non-catheterized patients, among the two studied groups.

	Catheter				
	BEEP		THULEP		
	Mean	Standard Deviation	Mean	Standard Deviation	P value
Qmax W1	26.15	3.93	27.07	3.99	0.553
Qmax MO1	29.92	3.52	27.79	3.68	0.137
Qmax Mo3	31.46	3.45	35.21	3.33	0.008
Qmax year	33.15	3.87	37.43	3.94	0.009
PVR W1	43.46	5.99	30.79	2.39	<0.001
PVR MO1	31.54	2.26	22.14	6.07	<0.001
PVR MO3	20.92	5.39	15.50	5.10	0.013
PVR 1 year	16.54	4.88	14.64	3.95	0.276
	No catheter				
	BEEP		THULEP		
	Mean	Standard Deviation	Mean	Standard Deviation	P value
Qmax pre (ml/sec)	6.75	2.01	7.31	2.26	0.208
Qmax W1	26.51	4.32	27.28	3.90	0.369
Qmax MO1	30.45	4.32	32.15	4.71	0.072
Qmax Mo3	32.53	5.48	35.78	4.09	0.002
Qmax year	34.00	5.26	38.09	4.04	<0.001
PVR pre	129.85	25.40	124.17	24.48	0.275
PVR W1	32.23	10.34	33.50	9.82	0.546
PVR MO1	19.62	3.66	22.28	5.04	0.005
PVR MO3	16.51	5.77	16.41	3.72	0.923
PVR 1 year	12.57	4.20	11.85	3.75	0.382

There were significantly lower urinary tract symptoms (LUTS) and dysuria in ThuLEP (60% of patients) compared to 25% in patients of bipolar group (*p* = 0.001), these symptoms improved significantly within 3 months.

After 1 month, urgency urinary incontinence was reported in 34 (56.7%) cases in ThuLEP and 14 (23.3%) cases in bipolar enucleation group (*p* value 0.001). Stress urinary incontinence was recorded in 44 (73.3%) cases in ThuLEP and 26 (43.3%) cases in bipolar enucleation group, respectively (*p* value 0.001). were managed by anticholinergics for urge incontinence cases, and performing Kegel’s exercise for SUI cases. Both types of incontinence improved after 3 months in both groups. After 1 year, three cases in the ThuLEP group had persistent stress incontinence, but all cases in the bipolar group recovered from stress incontinence (p = 0.2) (Table 4).

**Table 4.** Post-operative urinary incontinence.

Parameter		1 month	3 months	6 months	12 months
**Urgency**	Bipolar	16 (26.7%)	12 (20.0%)		
	ThuLEP	39 (65.0%)	5 (8.3%)		
	P value	<0.001	0.067		
Urgency incontinence	Bipolar	14 (23.3%)	6 (10.0%)		
	ThuLEP	34 (56.7%)	5 (8.3%)		
	P value	<0.001	0.752		
Stress incontinence	Bipolar	26 (43.3%)	9 (15.0%)	5 (8.3%)	0 (0.00%)
	ThuLEP	44 (73.3%)	28 (46.7%)	11 (18.3%)	3 (5%)
	P value	0.001	<0.001	0.107	0.236

UI: urgency incontinence, SUI: stress urinary incontinence, MO: month.

ICIQ-SF was used to assess SUI postoperatively, in the BEEP group, the mean scores for ICIQ at 1 month, 3 months, and 1 year were 0.73, 0.30, and 0.00, respectively. In comparison, the THuLEP group had mean scores of 2.80, 0.68, and 0.13 for ICIQ at 1 month, 3 months, and 1 year, respectively. The *p* values for the comparisons between the BEEP and THuLEP groups were <0.001 for ICIQ Mo1, 0.083 for ICIQ MO3, and 0.088 for ICIQ 1 year (Table 5).

**Table 5.** ICIQ-Sf at 1 m,3 m, and 1 year between both groups.

	BEEP					THULEP					
	Mean	SD	Median	Minimum	Maximum	Mean	SD	Median	Minimum	Maximum	P value
ICIQ Mo1	0.73	2.00	0.00	0.00	8.00	2.80	2.89	2.00	0.00	11.00	<0.001
ICIQ MO3	0.30	1.05	0.00	0.00	5.00	0.68	1.33	0.00	0.00	5.00	0.083
ICIQ 1 y	0.00	0.00	0.00	0.00	0.00	0.13	0.60	0.00	0.00	3.00	0.088

The ICIQ-UI SF is divided into the following four severity categories: slight (1–5), moderate (6–12), severe (13–18) and very severe (19–21).

On the other hand, there is a significant effect of pre-operative prostate size on post-operative one-month urgency in BEEP group only with p-value = 0.043, EXP B = 1.043, which means that with each change in prostate size by one-unit results in an increase in urgency by 4.3% (when other factors were constant).

No significant differences were observed as regard intraoperative and postoperative complications. There were mild intraoperative complications (subtrigonal injury, small insignificant capsular perforation and mild bladder mucosal injury by the morcellator) which did not need further intervention, nor prolongation of catheterization time, and one case in ThuLEP group and two cases in bipolar group developed postoperative secondary haematuria and were treated conservatively and needed blood transfusion but didn’t need cystoscopy (Clavien grade IIa). Febrile urinary tract infection occurred in two cases of both groups (Clavien grade IIa). One case in each group developed epididimo-orchitis. Bladder neck contracture occurred in one case of ThuLEP and was treated by bladder neck incision (Clavien grade IIIa). Urethral stricture occurred in one case of bipolar group requiring visual urethrotomy (Clavien grade IIIa). (Table 2)

## Discussion

Endoscopic enucleation of the prostate has been the gold standard treatment for bladder outlet obstruction with a large prostate since 2016 [11].

In 2019, a met-analysis done by Huang SW et al., evaluating nine surgical treatments for BPO included 109 trials with a total of 13 676 patients and concluded that enucleation methods had better Qmax and IPSS values than vapourization and resection [12]. The adaptation of prostatic enucleation technique is boosted by the familiarity with the finger assisted anatomical enucleation of the adenoma during open prostatectomy.

In the last few years, safety and efficacy of THuLEP and BEEP had been established in comparison to the gold standard TURP and also the against the more popular enucleation technique HOLEP, but comparative studies regarding peri-operative data are still scarce.

Thulium-YAG laser serves as an exceptional energy source for endoscopic prostatic enucleation, providing a clear and bloodless incision in prostatic tissue. The concept of anatomical enucleation utilizing thulium energy through extensive blunt dissection of the adenoma was first introduced by Herrmann et al. in 2010 [4]. This approach of blunt, energy-free, enucleation reduces the risk of capsular perforation, limits energy scattering to pericapsular tissues, and alleviates postoperative irritative symptoms [12].

Transurethral bipolar (plasma-kinetic) enucleation of the prostate was first described in 2004 [11]. A 2006 randomized controlled study concluded that bipolar enucleation (BEEP) was equivalent to HOLEP [13]. Additionally, two meta-analysis studies in 2015 confirmed the efficacy of this enucleation technique [14,15]. In 2016, the European Association of Urology guidelines recognized both HOLEP and bipolar enucleation as the primary treatments for obstructive symptoms caused by a large prostate (over 80 gm).

Iacono et al. studied the functional outcomes of ThuLEP (120 W/40 W) in 148 patients and found a significant improvement of all parameters [16]. Similarly, Gross AJ et al. in his prospective study on 1080 patients undergoing ThuVEP found a significant improvement of all parameters in all groups [17]. Netsch C et al. found the same results in their prospective study included 124 ThuVEP patients [18].

In a systemic review and meta-analysis comparing ThuVEP/ThuLEP (TmLRP) with TURP, the improvement of Qmax and IPSS favored ThuVEP/ThuLEP while PVR and QoL were not significantly different [19]

On the other side, Chunxiao Liu et al. studied the effectiveness of bipolar enucleation (BEEP) in a series of 1.100 patients with a median follow-up of 4.5 years. They found a significant improvement of all parameters [20]. Lingfeng Zhu et al. compared bipolar EEP vs. TURP in a randomized clinical trial (RCT) including 80 patients and demonstrated the same improvement of urinary symptoms [21].

Arcaniolo et al., in their meta-analysis of 14 comparative studies involving 2317 patients (1178 undergoing BEEP and 1139 undergoing bipolar TURP), found no differences in operative time between the two procedures. However, BEEP resulted in a higher amount of resected tissue, shorter bladder irrigation duration, shorter hospital stays, and reduced catheterization times, demonstrating clear superiority of BEEP in terms of functional outcomes [22].

In a meta-analysis done by Gu C et al. in 2020 included 31 published articles (4382 patients), comparing laser treatment with bipolar technology, safety and efficacy profiles were comparable between bipolar and laser treatments. With less Hb drop, shorter catheterization duration and shorter hospital stay in lasers groups. However, the smaller reduction in Hb, with lasers, did not translate into a reduced transfusion rate [23].

Habib et al., Higazy et al. and Neill et al. found that HOLEP procedure was associated with shorter operative and enucleation time than bipolar enucleation, but we did not find this difference in our study between ThuVEP and BEEP [13,24,25]. Higazy et al. found shorter hospital stay and catheterization time in HOLEP than BEEP but Habib et al. and Neill et al. did not find these differences similarly to our result in this study.

Gamal Eldin A et al. in 2024 Evaluated the early apical release with bipolar Collins knife versus THuLEP for large-sized prostate and found that: early apical release was safe and effective with significant decrease in post-operative stress incontinence during early follow-up. Intraoperative irrigation volume, and post-operative Hb drop favored the bipolar group [26].

In a 2024 prospective analysis conducted by Abdelaziz et al. on thulium laser enucleation for benign prostatic hyperplasia, comparing low and high-power approaches for prostates exceeding 80 g, it was observed that irritative symptoms (namely urgency and dysuria) significantly differed between the two groups in the first 3 months (50% in the high-power group versus 15% in the low-power group) with a *p* value of 0.001. However, these symptoms did not persist beyond 6 months, aligning with our findings using high-power thulium YAG laser [27].

A retrospective analysis done by Hirasawa et al. on 584 patients who underwent bipolar enucleation of the prostate found postoperative transient urine incontinence in 17.3%, 13.5%, 3.1%, 0.41%, and 0%, at 1, 3, 6, and 12 weeks, respectively, where age and prostate volume were significant factors [28].

In our study, there were no notable differences between the two groups concerning intraoperative and postoperative complications. According to the study by Iacono et al., only 2.7% of patients who underwent ThuLEP required early post-surgery blood transfusions due to ongoing hematuria. Additionally, urinary tract infections occurred in 12.8% of patients, and two patients developed urethral stricture, which was treated with cold incision [17]. In a study by Chunxiao Liu et al., meatal stenosis was found in 9 out of 1100 BEEP cases. Urinary incontinence was observed in 56 patients, while urethral stricture affected 12 individuals. Additionally, only 10 patients experienced bladder neck contracture over a 6-year follow-up period [21]. In a study by Gross AJ et al., it was reported that the most common early complication in ThuVEP cases was urinary retention, occurring in 9% of patients. Re-intervention, including repeat morcellation, secondary apical resection, and clot removal, was necessary for 4.7% of patients. Hemorrhage requiring blood transfusion was observed in 1.7% of cases, and it was noted that the complication rate was dependent on prostate size [18].

## Conclusion

Both ThuLEP and BEEP are effective and safe techniques for the treatment of benign prostatic hyperplasia (BPH) in prostates exceeding 80 g. While ThuLEP showed some perioperative advantages, such as higher Qmax values and lower LUTS, yet high power can cause higher rates of SUI in early postoperative period both techniques demonstrated comparable functional results and complication rates. Larger-scale studies with extended follow-up periods are recommended to further compare the overall outcomes of these techniques.

## References

[1] Gupta N, Anand A. Comparison of TURP, TUVRP, and HoLEP. Curr Urol Rep. 2009;10(4):276–278. doi: 10.1007/s11934-009-0045-4 19570488

[2] Hiraoka Y, Lin T, Tsuboi N, et al. Transurethral enucleation of benign prostatic hyperplasia. Nihon Ika Daigaku Zasshi. 1986 Apr;53(2):212–215. doi: 10.1272/jnms1923.53.212 2423551

[3] Bach T, Wendt-Nordahl G, Michel MS, et al. Feasibility and efficacy of Thulium: YAG laser enucleation (VapoEnucleation) of the prostate. World J Urol. 2009;27(4):541–545. doi: 10.1007/s00345-008-0370-0 19184038

[4] Herrmann TRW, Bach T, Imkamp F, et al. Thulium laser enucleation of the prostate (ThuLEP): transurethral anatomical prostatectomy with laser support. Introduction of a novel technique for the treatment of benign prostatic obstruction. World J Urol. 2010;28(1):45–51. doi: 10.1007/s00345-009-0503-0 20063164

[5] Morsy S, Elfeky M, Abdel-Rahman S, et al. Comparing surgical techniques: ThuLEP and transurethral BPEP for prostate over 80 grams. Intraoperative and postoperative results. A prospective randomized trial. Arab J Urol. 2024;23(1):1–7. doi: 10.1080/20905998.2024.2395594 39776558 PMC11703526

[6] Enikeev D, Glybochko P, Rapoport L, et al. A randomized trial comparing the learning curve of 3 endoscopic enucleation techniques (HoLEP, ThuFLEP, and MEP) for BPH using mentoring approach—initial results. Urology. 2018;121:51–57. 30053397 10.1016/j.urology.2018.06.045

[7] Pirola GM, Saredi G, Codas Duarte R, et al. Holmium laser versus thulium laser enucleation of the prostate: a matched-pair analysis from two centers. Ther Adv Urol. 2018;10(8):223–233. doi: 10.1177/1756287218779784 30034541 PMC6048626

[8] Mallikarjuna C, Nayak P, Ghouse SM, et al. Transurethral enucleation with bipolar energy for surgical management of benign prostatic hyperplasia: our initial experience. Indian J Urol. 2018 Jul-Sep;34(3):219–222. doi: 10.4103/iju.IJU_71_16 PMID: 30034134; PMCID: PMC6034404. 30034134 PMC6034404

[9] Chan YH. Biostatistics102: Quantitative Data – Parametric & Non-parametric Tests. Singapore Med J. 2003;44(8):391–396. 14700417

[10] Chan YH. Biostatistics 103: qualitative data –tests of independence. Singapore Med J. 2003;44(10):498–503. 15024452

[11] Herrmann TRW, Gravas S, de la Rosette JJ, et al. Lasers in transurethral enucleation of the prostate—Do we really need them. J Clin Med. 2020 10;9(5):1412. doi: 10.3390/jcm9051412 32397634 PMC7290840

[12] Huang SW, Tsai CY, Tseng CS, et al. Comparative efficacy and safety of new surgical treatments for benign prostatic hyperplasia: systematic review and network meta-analysis. BMJ. 2019 Nov;14(367):l5919. doi: 10.1136/bmj.l5919 PMID: 31727627; PMCID: PMC7223639.Need Them J Clin Med. 2020; 10;9(5):1412. PMC7223639 31727627

[13] Lin Y, Wu X, Xu A, et al. Transurethral enucleation of the prostate versus transvesical open prostatectomy for large benign prostatic hyperplasia: a systematic review and meta-analysis of randomized controlled trials. World J Urol. 2015;34(9):1207–1219. doi: 10.1007/s00345-015-1735-9 26699627

[14] Li M, Qiu J, Hou Q, et al. Endoscopic enucleation versus open prostatectomy for treating large benign prostatic hyperplasia: a meta-analysis of randomized controlled trials. PLOS ONE. 2015;10(3):e0121265. doi: 10.1371/journal.pone.0121265 25826453 PMC4380430

[15] Xiao K-W, Zhou L, He Q, et al. Enucleation of the prostate for benign prostatic hyperplasia thulium laser versus holmium laser: a systematic review and meta-analysis. Lasers Med Sci. 2019;34(4):815–826. doi: 10.1007/s10103-018-02697-x 30604345

[16] Iacono F, Prezioso D, Di Lauro G, et al. Efficacy and safety profile of a novel technique, ThuLEP (Thulium laser enucleation of the prostate) for the treatment of benign prostate hypertrophy. Our experience on 148 patients. BMC Surg. 2012;12(Suppl 1):S21. doi: 10.1186/1471-2482-12-S1-S21 23173611 PMC3499280

[17] Gross AJ, Netsch C, Knipper S, et al. Complications and early postoperative outcome in 1080 patients after thulium vapoenucleation of the prostate: results at a single institution. Eur Urol. 2013;63(5):859–867. doi: 10.1016/j.eururo.2012.11.048 23245687

[18] Netsch C, Engbert A, Bach T, et al. Long-term outcome following Thulium vapo-enucleation of the prostate. World J Urol. 2014;32(6):1551–1558. doi: 10.1007/s00345-014-1260-2 24531878

[19] Zhu Y, Zhuo J, Xu D, et al. Thulium laser versus standard transurethral resection of the prostate for benign prostatic obstruction: a systematic review and meta-analysis. World J Urol. 2014;33(4):509–515. doi: 10.1007/s00345-014-1410-6 25298242

[20] Liu C, Zheng S, Li H, et al. Transurethral enucleation and resection of prostate in patients with benign prostatic hyperplasia by plasma kinetics. J Urol. 2010;184(6):2440–2445. doi: 10.1016/j.juro.2010.08.037 20952005

[21] Zhu L, Chen S, Yang S, et al. Electrosurgical enucleation versus bipolar transurethral resection for prostates larger than 70 mL: a prospective, randomized trial with 5-year followup. J Urol. 2013;189(4):1427–1431. doi: 10.1016/j.juro.2012.10.117 23123549

[22] Arcaniolo D, Manfredi C, Veccia A, et al. Bipolar endoscopic enucleation versus bipolar transurethral resection of the prostate: an ESUT systematic review and cumulative. World J Urol. 2020;38(5):1177–1186. doi: 10.1007/s00345-019-02890-9 31346761

[23] Gu C, Zhou N, Gurung P, et al. Lasers versus bipolar technology in the transurethral treatment of benign prostatic enlargement: a systematic review and meta-analysis of comparative studies. World J Urol. 2020 Apr;38(4):907–918. doi: 10.1007/s00345-019-02852-1 Epub 2019 Jun 17. PMID: 31209562.analysis. World J. Urol. 2019. 31209562

[24] Habib E, Ayman LM, ElSheemy MS, et al. Holmium laser enucleation vs bipolar plasmakinetic enucleation of a large volume benign prostatic hyperplasia: a randomized controlled trial. J Endourol. 2020;34(3):330–338. doi: 10.1089/end.2019.0707 31813283

[25] Higazy A, Tawfeek A, Abdalla H, et al. Holmium laser enucleation of the prostate versus bipolar transurethral enucleation of the prostate in management of benign prostatic hyperplasia: a randomized controlled trial. Int J Urol. 2021;28(3):333–338. doi: 10.1111/iju.14462 33327043

[26] Gamal Eldin A, Abdallah M, Fouad A, et al. Evaluation of early apical release with bipolar Collins knife versus Thulium-Yag laser enucleation of large-sized prostate. A randomized study. Arab J Urol. 2024 Feb 23;22(3):179–185. doi: 10.1080/20905998.2024.2321737 PMID: 38818261; PMCID: PMC11136459. 38818261 PMC11136459

[27] Abdelaziz AY, Kamal I, Abdelhakim MA, et al. A prospective analysis of thulium laser enucleation in benign prostatic hyperplasia comparing low- and high-power approaches for prostates exceeding 80 g. World J Urol. 2024 Apr 27;42(1):265. doi: 10.1007/s00345-024-04901-w PMID: 38676756; PMCID: PMC11055731. 38676756 PMC11055731

[28] Hirasawa Y, Kato Y, Fujita K. Age and prostate volume are risk factors for transient urinary incontinence after transurethral enucleation with bipolar for benign prostatic hyperplasia. Int J Urol. 2017;25(1):76–80. doi: 10.1111/iju.13472 28975723

